# How to Provide Sexual Education: Lessons from a Pandemic on Masculinity, Individualism, and the Neoliberal Agenda

**DOI:** 10.3390/ijerph18084144

**Published:** 2021-04-14

**Authors:** Sharon Lamb, Marta Pagán-Ortiz, Sara Bonilla

**Affiliations:** 1 Counseling and School Psychology, College of Education and Human Development, University of Massachusetts Boston, Boston, MA 02125, USA; sara.bonilla001@umb.edu; 2 Cambridge Health Alliance, Cambridge, MA 02139, USA; mpaganortiz@challiance.org

**Keywords:** sex education, neoliberalism, pandemic, health, citizenship, condoms, masculinity, individualism, collectivism

## Abstract

Sex education in the United States is often approached through an individual lens that focuses on personal protection, safety, and rights. This focus on personal responsibility and care-for-self reflects national values and permeates governmental systems and actions, including generalized public health approaches. This issue has been most recently highlighted in the individual and systemic attitudes, beliefs, and responses towards the recent, ongoing crisis following the global surge of COVID-19. In this paper, we provide examples and discuss lessons gleaned from the public health response to this crisis, particularly in the areas and intersections of gender, individualism, and neoliberalism, and the parallels of these issues in sex education. We make an appeal for a collectivist and community-oriented approach to sex education, which would focus not only on prevention and protection, but on inequities, ethics, and care for others.

## 1. Introduction

Sexual education is a public health issue, even if it hasn’t been prioritized as such in the United States (where we write from). In the United States, sexual education has typically been taught within a primary public health prevention framework as a way to prevent pregnancy and sexually transmitted infections (STIs) [1]. We have argued in other papers [2,3] for an expansion of sex education beyond this general prevention model. Our work has proposed that sex education needs to encompass approaches that specifically seek to develop students into “sexual citizens,” teaching them values with regard to how the expression of individual sexual desires impacts other people and giving them the opportunity to discuss cultural issues around sex and sexualities. This latter approach is generally not viewed as essential to public health, but arguably does in fact address public health concerns relating to the prevention of sexual violence, promotion of psychological well-being, and a healthier citizenship. This rare approach works through an overarching ethics-centered, communitarian-focused approach. Sex education, as it is most often taught, focuses on individual students, asking them to understand risk to self and ways to protect themselves from sexual ills (defined for the most part as pregnancy and disease, and sometimes, simply early sexual activity) [4]. In short, this is an individualized protection approach which aims to change students one by one and create greater personal responsibility via self-care.

The problems inherent to an individualized approach to public health via self-protection and the subsequent focus on personal rather than group responsibility have been made increasingly salient during the recent global crisis following the surge of COVID-19. During this time, citizens have been called upon to make sacrifices for the good of their communities, and to reframe what would seem to them to be self-protective measures as ways of protecting others and community. There are many parallels between the current state sex education and the response to this pandemic, such as the education and appeals around condom and mask-wearing. There are also deeper commonalities in regard to what it means to be a part of a community, and what it means to have others’ welfare in mind as one simultaneously pursues individual needs and protections. This paper focuses on the overlap between sexual education and public health responses to COVID-19 (understanding that this education has come in the form of public announcements rather than specific curricula). We discuss parallels between these two issues, particularly related to gendered responses and resistances to thinking collectively. We also call on theories regarding an “ethic of care” and communitarianism which run against a neoliberal approach to public health education.

We first address the parallels between sex education and the response to the pandemic by discussing the rugged individualism prized in the United States—a way of relating to self and others that has deep connections to U.S. history, masculinity, and capitalism [5]. Individualism constitutes an institutionalized set of values that prioritizes autonomy, individual rights, freedom, and choice. It is contrasted to other ways of being and value systems, such as collectivism which is more valued in other countries and even cultures within the U.S. It is also contrasted to a care ethics approach [6,7] which prioritizes care for others as a central moral motivation. In the U.S., the culture of individualism is expressed in its extreme form through libertarianism and neoliberalism wherein laws and policies are developed to benefit and protect individuals over communities, following the belief that asking for sacrifices from the community is an impingement on individual liberty and that individuals are responsible for their own outcomes. The existence of neoliberalism requires a blindness to social conditions and an unsubstantiated belief that everyone has the ability and the access to make individual decisions [8].

In the middle of a pandemic, when the former President scoffed at the use of masks and minimized the dangers of a virus (e.g., asking his followers and team to meet without masks, and mocking those who are afraid) [9], we ask whether lessons learned about the limits to individualism might be applied to sex education. Although at the time of publication, the crisis may be controlled by the rollout of vaccines, we believe the lessons about individualism that current responses have taught us should provide important information for sexual education in the future. After examining the current response to the pandemic, we make comparisons to sex education as it is currently configured in the U.S. and draw lessons for the future.

## 2. Masculinity and COVID

It is essential to discuss the connection between individualism and hegemonic masculinity as it affects individuals’ and the public’s responses to the pandemic, as well as the way sexual education can be configured in curricula for students. The concept of hegemonic masculinity has undergone changes since its initial introduction by Connell [10,11]. Hegemonic masculinity refers to a “set of values, established by men in power that functions to include and exclude, and to organize society in gender unequal ways” [12], (p. 40). The concept can encompass individualism, power over others, and an entitlement to break the rules. Rule-breaking is embedded in the U.S.’s history such that questioning and bucking authority, and celebrating mavericks have become central to U.S. masculinity [5]. 

The impact of masculinity on men’s health and health-seeking behaviors has been a subject of study for over four decades [13,14]. In relation to men and health, Jewkes and colleagues write “The system that keeps men in a collectively dominant position over women and in competitive relations to other men comes at a cost for men in terms of their health and quality of life. Faced with an ideal where physical resilience is valorised, men find it harder to seek healthcare and engage in preventive activities” [15].

Hegemonic masculinity is characterized by risk-taking health behaviors [16], for example, in alcohol consumption, smoking, and now pandemic risk. Masculinity influences sexual risk-taking, avoidance of health clinics [17], and, of course, women’s health through promotion of violence against women, and, historically, the spread of infectious disease to wives and sex workers through male philandering [18].

With regard to pandemic risk, a recent report finds that the “influence of masculinist perspectives has hampered government responses to coronavirus, encouraging dismissiveness based on notions of being ‘too tough’ to need to worry about it and the use of warlike rhetoric in framing the pandemic” [19]. Thomson [20] discusses how leaders, particularly Trump, have called on hegemonic masculinity to respond to the pandemic. They write that the response to the pandemic required acknowledging risk and taking measures to prevent which were seen as “feminine” and “weak.” An increasingly “macho” U.S. context played a role in the government’s inadequate response. These authors also note that a masculinist perspective not only hurts the general population but is particularly harmful to girls and women. Women, as the primary caregivers of the young and elderly, have additional responsibilities to others; women are more likely to lose their jobs than men; and that, during “lockdown” phases, men’s violence towards women increases.

This masculinist approach to the pandemic has resulted in higher rates of death among men, and greater levels of male violence in the home [19]. It extended from average everyday refusals to wear masks to extremist plots to kidnap female Governor Gretchen Whitmer of Michigan for enforcing “tough lockdown measures” [9]. Before relating this masculinist perspective to sex education, we discuss the general public health response to the pandemic in hopes of showing parallels to the failure of sex education.

## 3. Public Health Response to the Pandemic

Notwithstanding two centuries of manifest masculinity in the U.S., there is a great temptation to be surprised by all of this—the confusion and derision with regard to simple scientific facts and directives in the public interest that continue to lead to more outbreaks and lack of containment of the virus. It is tempting to look at this outbreak and wonder, “How is it possible that this could happen here?” *Here*, referring to a purported vastly resourceful country in terms of capital wealth and education. It is similar to the shock displayed among certain groups towards the continuing high rates of teen pregnancy in the U.S. compared to other Western countries, and rates of sexual violence that stay constant in spite of numerous changes in awareness regarding what non-consensual sex is and how it affects those who are sexually victimized. We ask, how are these issues still prevalent despite the vast amounts of knowledge amassed, as well as the myriad array of tools available to address these concerns? At the base of addressing these issues, which are often understood in the context of their physiological (in the case of COVID-19) or individualized psychological impact (in the case of sexual assault), lie social answers. Below, we consider the following in order to later make parallels to sex education in the U.S.: the neoliberal promotion of individual freedoms; the crisis in leadership; and the invisibility or lack of care towards vulnerable populations.

### 3.1. The Prominence of Individualism

During the first months of the pandemic, the former government-in-charge relied on individual responses and behaviors as a way to quell infection rates. While there have been guidelines and regulations around schools, businesses, and other systems operations, these have been inconsistent across states, and even across counties. Handwashing, mask-wearing, and physical distancing are in fact key components to maintaining health; however, these are often heavily counted on, while plans to address large-scale issues (e.g., misinformation, school-related issues, inability to pay rent, inadequate bailouts for small businesses) have fallen by the wayside. Encouraging and relying on individual responses have been problematic for another reason: these are deeply personal, value-laden choices influenced by the much broader social issues the government has so ineptly and irresponsibly addressed.

There have been a number of scientific articles and opinion pieces addressing the issue of “American individualism” and the impact of this on virus containment. Briefly, these articles [21,22] explore the idea of so-called American freedom, liberty, and individual rights, and the impact of these ideas on a culture of rugged individualism in which people feel that their personal choice matters above all else. One author [23] keenly describes the concept of freedom as related to the pandemic:

*“In a society in which individual freedom is practiced with full awareness of the rights of others and the solidarity which underpins a mutually regarding and respectful community, it is natural to wear a mask during the pandemic—not to protect oneself but to block transmission from self to others. In a society in which the unconstrained individual always has primacy, the requirement that a mask must be worn, like the requirement that one must stay at home until permitted to leave, is less the regulation of common social protection than a violation of the person.”* (p.1393 ).

What the author reminds us of is that, while individualism can certainly correspond to the set of values held by any given person, in this case (and many other public health scenarios), it is a quality that is reinforced by broader forces. Take, for example, the multiple, contradictory messages and responses provided by various levels of government. In May 2020, the CDC issued a report that outlined some lessons learned and the public health priorities and responses at the beginning of the pandemic, February through April 2020 [24]. In this report, the authors outline the factors and events related to accelerated spread during those months, including: travel, large social events, limited testing, and density in workplace settings. The CDC issued guidance (albeit contradictory initially) for the use of cloth face coverings, and for the initial acceleration phase of the virus, they advocated for stay-at-home orders and the closing of schools and non-essential businesses. The report culminates with a final guidance, stating that these initial responses might continue to be needed in later phases of the outbreak.

But well before the CDC issued this report, as far back as February or March of 2020, it appeared that there would be significant challenges with compliance and care in the U.S. while many countries have successfully encouraged their citizens to follow the same individual directives (e.g., handwashing, mask-wearing, physical distancing). But the problem does not lie with the requests for compliance with individual behaviors, but instead lies with a lack of collective responsibility that in turn holds people accountable for collective failures. As mentioned, plenty of other countries, some with self-described “collectivist” cultures have asked their citizens to engage in these individual behaviors. However, (a) the messaging and the purpose of engaging in these behaviors has been different (i.e., care for others), (b) engaging in these behaviors has been accompanied by social, public policies which also reinforce the work towards a “greater good,” and (c) some have even enforced fines for lack of compliance. Current research based on global responses to the COVID pandemic has highlighted these differences between the U.S. and other countries and offered guidance. Studies have shown that individualistic responses in times of collective crisis give way to negative public health consequences [25].

In the U.S., it appeared that, even when given information that masks save *other people’s lives*, before it was also clear that they provide some protection for the self, individuals continued to respond to mask-wearing as if it were about self-protection. Those who refrained would say that they didn’t need to wear a mask because they were young, and felt they would thus be unaffected, or that they were not afraid (which indicated they believed the mask was protecting themselves and not others). Leaders referred to their own feelings of safety which undermined the scientific understanding that we wear masks to prevent *others* from getting the virus in case we are asymptomatic transmitters. Indeed, early on, it was clear that the way the virus was spreading, (e.g., the Biogen conference, a “super-spreader” event in Boston, 26–27, February, 2020, was found to have happened through mostly asymptomatic carriers [9].

### 3.2. A Crisis in Leadership

In a democratic regime like the United States, some authors [26,27] have suggested that, while it might be harder to enforce compliance to certain behaviors, the public could “benefit from better information flow and public trust” [26]. During the former presidential administration, rife with misinformation and denialism, public trust in leadership has been severely polarized. A recent paper on crisis leadership outlined clear strategies to mitigate risk during the pandemic [27,28]. At the very least, the United States’ federal response does not meet most of the thirteen strategies, developed based on existing work and literature around leadership in crisis and risk management [27]. The strategies include transparency, empathy, solidarity, direction giving, and partnership, all of which require a sense of community care.

One factor which influenced how and why these guidelines were disregarded is the varying degrees of power of local, state, and federal authorities. While the CDC, a government entity, is delivering guidance around how to mitigate transmission, the country also had multiple high-level politicians (including the former President) showing disdain at these directives. Politicians all over the country showed disregard for mask-wearing, the closing of non-essential businesses, and for scientific counsel. Airports remained open, tourism boards continued encouraging travel, non-essential settings remained open in order to continue to keep small businesses and the overarching economy afloat. Restaurants held and continue to offer indoor dining. Political campaigns held large rallies with little to no attention paid to protection, including the Rose Garden celebration of the appointment of Amy Coney Barret, an event Dr. Anthony Fauci has suggested was a superspreader. After the former President’s own COVID-related hospitalization, his message was clear as he ripped off his mask: “Don’t be afraid of COVID …. Don’t let it dominate your life” [9]. All of these events implied that individuals had to make their own choices. The “live your life” narrative is one that implies being “brave,” “adventurous,” and not risk-averse—a narrative mostly embraced by men [29]. Rather than focusing on system-wide measures, which imply that everyone should be cared for equally, these higher-level responses suggested: “everyone needs to take action to take care of themselves.”

### 3.3. Ignoring Vulnerable Populations

Given that individual behaviors and choices have been the primary line of protection and care, people are invited to and inclined to make decisions that are best for them and their family, with little to no consideration of the impact of these decisions on others. One example includes putting parents in insidious binds, in which they have to choose between a choice that aligns with their communal values (e.g., supporting public education) and a choice that relates to their individual wellbeing (e.g., taking their child out of school in order to keep them safe/healthy). This response also highlights the issue of so-called choice—and what is available to some and not to others.

In essence, this response has been remiss in the way COVID affects people of color differently, as well as populations from other marginalized groups. More recently, researchers have begun to label the current crisis as a syndemic in the United States, not a pandemic, as it is most commonly called [30,31]. The concept of syndemic takes into account how sociopolitical factors engage with a disease—how, for example, these factors can mitigate or worsen the propagation of a disease. For example Horton writes,

*“The most important consequence of seeing COVID-19 as a syndemic is to underline its social origins. The vulnerability of older citizens; Black, Asian, and minority ethnic communities; and key workers who are commonly poorly paid with fewer welfare protections points to a truth so far barely acknowledged—namely, that no matter how effective a treatment or protective a vaccine, the pursuit of a purely biomedical solution to COVID-19 will fail [31]”* (p. 874).

In the U.S., issues such as systemic racism and a broken, inaccessible healthcare system, which focuses on treatment and not prevention, have exacerbated the emergence of COVID-19, particularly in vulnerable populations. By vulnerable populations, we are specifically referring to minoritized ethnic and racial groups, which are at increased risk of getting COVID and receiving less-than-ideal care and treatment for it. These social factors, including discrimination, access to care, and occupations, have left these groups of people with little choice in terms of individual behaviors to self-care [32]. Occupations and wealth gaps have left individuals with no choice in capacity for distancing and/or working remotely, and little choice in terms of accessing care. As they are left to advocate and care for themselves, they are additionally deemed “heroes” by the media and the public at large. They are the workers who go in to do service and are “heroes” because they go above and beyond what is expected or what people with more privilege would do. If we had a culture where we were expected to care for the vulnerable, then we wouldn’t need to have heroes at all… it would be part of the way we operate as a nation.

## 4. Parallels to Sex Education: Individualism; Crisis in Leadership; Vulnerable Populations

There is widespread agreement that the U.S. failed to contain the coronavirus when it could, in the early stages [33]. Nevertheless, there were geographic areas with strong leadership and community buy-in that had fewer cases and ultimately fewer deaths [34]. Similarly, sex education in the U.S. has, for the most part, failed, resulting not only in unwanted pregnancies and STIs, but the elevation of pornography as a means of learning about sex with its concomitant sexism, aggression, and focus on harmful practices, and the continued high rates of sexual assaults [35,36,37,38,39]. Few political figures are willing to stick their neck out on this issue and many continue to put forward misinformation just like President Trump did around the pandemic (e.g., that condoms are not effective, abortions are unsafe; and rape is perpetrated by sick people who jump out of the bushes and attack women who walk alone at night) [40]. We discuss the parallels between the approach to the virus and the approach to sex education, in the U.S., below via the same three foci we introduced above: individualism; crisis in leadership; and vulnerable populations.

### 4.1. The Prominence of Individualism

When advocates of sex education speak of sex education protecting individual students from sexually transmitted diseases, pregnancy, and other harms of sex, they are promoting a personal responsibility perspective. Indeed, President Obama’s response to the decades-long rule of Abstinence Only Until Marriage curricula was through the introduction of the Personal Responsibility Education Program. To the extent that curricula do not talk about what we as a nation expect sex education to be, how to teach students to protect others as well as themselves, how to give pleasure as well as receive it, how sexual problems affect the people, neighborhoods, and groups they belong to, and how to think about their contributions to sexual problems in society (sex without consent; bullying; unfettered access to damaging pornography, bystanding, etc.), they buy into what some have called a neoliberal perspective [8,41,42,43]. While it makes sense that with an area so personal as sex, an adolescent might need to assume personal responsibility for their decisions, all too often, personal responsibility is translated into a discourse of refraining from risk-taking as a way to preserve one’s own future opportunities.

The benefits of sex education to the individual are understood. Good programs are connected to students delaying initiation of sexual intercourse, having fewer sex partners, fewer experiences of unprotected sex, more protected sex, and better grades [4]. But adolescents should also have “good” sex (ethical sex) with a sense of belonging to health-aspiring schools and communities and in order to protect other people and not only themselves. We see this focus for other aspects of well-being taken up by schools that emphasize community. For example, it is wrong to bully another student not only because it harms the other student, but also because it harms groups of students who identify with the harmed student, and even more importantly, the school.

Why the individual focus? Perhaps most importantly with regard to the connections between sex education and the pandemic response, we should entertain how neoliberalism has invaded the approach to sex education. Neoliberalism is an ideology influencing economic and social policy that advocates for individual freedoms and personal responsibility, and curbs regulations and social welfare to advance capitalism [41,44]. It serves in opposition to “the collective society” and “has perverse consequences for social and political life” [44]. In promoting freedom, neoliberals appear to be applying rules and regulations equally to all, but by assuming all start on an equal basis, and ignoring background conditions and access to resources and opportunities, they reinstate existing inequities under the guise of just deserts. With no single civic identity and only individuals, humans who are social animals, “retreat to tribalism and identity groups”; civic associations are replaced by other affiliations [44]. Hence, the paradoxical rugged individualism of the #MAGA groups who refused to wear masks.

Sinnika Elliott [43] showed the neoliberal approach in the discourse of sex education teachers. According to Elliott [43] who drew from Foucault, policies around sex education are “tied to larger governmental attempts to regulate populations, attempts that are infused with racialized, classed, gendered, and sexualized meanings and inequalities” (p. 211). Approaching sex from a libertarian/neo-liberal view that everyone is responsible for themselves (parents for their own children) and ethical sex results from minimal interference with personal and parental freedom means providing sex education in a way that teaches teens to be responsible for themselves. Not mandating it at all suggests that parents should assume the responsibility for educating their own children.

The neoliberal understanding of freedom, in sex education, influences policy in three additional ways. First, states and even individual districts are permitted to make their own decisions and the U.S. government is loath to intervene. Second, the argument that school-based sex education interferes with parents’ freedoms to raise their children according to their own values is trotted out over and over again. In other words, if neoliberalism doesn’t encourage or acknowledge any collective or civic affiliation, or even an affiliation with their local school, parents are free to find their own way to educate their children. Third, the way neoliberalism promotes freedom around issues of sex and sexuality is not in the sense that freedom comes from a good education, and towards that end, adolescents ought to have free access to information and services. Instead, it focuses on a sense of freedom that is boiled down to no restrictions. Adolescents are thus free to learn about sex from their parents, their peers, or the internet, without restriction. To educate them would be, to the neoliberal, an imposition of values.

We see this most clearly in the way neoliberalism supports the business of pornography demanding no restrictions or restrictive access, while also preaching that self-restraint is good in relation to pornography. But the burden is placed on the parents to restrict the child and the child to restrict themselves from access. The quintessential burden is on the individual when the market is free. Ignoring research on the effects of pornography on adolescents’ views of sex [45] just as mask avoiders ignore research on the health benefits to the community as well as to the individual, the neoliberal position on pornography prioritizes free markets over protection of a group of vulnerable people—adolescents. Ignoring pornography and focusing on pregnancy prevention, it is still the individual’s responsibility or the individual family’s responsibility to prevent teen sex. And children who get pregnant or end up with STIs, who coerce others into sex, or have problems with addictive sexual behaviors including overuse of pornography are themselves blamed.

The neoliberal approach may be embedded in the curriculum or expressed through the way schools carry out the curricula [43,46,47,48]. Blaming of the individual is clear in school-based sex education to the extent that the focus is on teaching students about risks and giving them strategies and tools to reduce risk in needed moments, “you play, you pay” [43]. But it would be wrong to think that this neoliberal approach is only present in the most conservative or abstinence-only curricula. Conservatives imploring teens to use self-restraint to be abstinent and progressives asking teens to use condoms; the former using values arguments, the latter science, are both in one sense doing the same thing—they are making their pleas to the individual student, placing the responsibility for self-protection on their individual shoulders. Failures thus are not the failure of the community, or the curriculum, or even the school as a collective as much as it belongs to the individual student who has taken the risk.

The blaming of individual teens is not the only outcome of neoliberal attitudes towards sex education. As with the pandemic, it is important to see who gets harmed the most in these situations and who faces the consequences. When teens get pregnant in richly resourced areas, where contraceptive access and sex education may be more available, there are opportunities and resources to work with the teen, even if she is still blamed for her pregnancy. Girls of color who become pregnant are positioned as oversexed and underparented [47], adultified, as they have been in the school system when they have gotten “in trouble.”

As with pandemic-think, it’s not the boy who doesn’t use contraception that is held responsible, or the person who refused to wear a mask, but the person who “catches” the virus or pregnancy for not protecting herself. The idea that sex education should teach taking care of another person is parallel in the pandemic wherein it was very difficult for individuals to understand that wearing a mask protects someone else. It’s a personal choice. But in sex education and in the pandemic, wearing “protection” protects others, your partner within a sexual relationship, your community with regard to the spreading of the virus or advancement of a group. As it is a personal choice to wear or not wear masks or condoms, the primary choice is about whether or not you will be thinking about, even caring about, other people and see their future as wrapped up in one’s own. And that is not a value of neoliberalism.

### 4.2. A Crisis in Leadership

As with COVID-19, there has been little agreement among the different U.S. states regarding what kind of sex education there should be, what is included in it, and whether it should be mandated. While many agree that sex education is crucial for the prevention of pregnancy and sexually transmitted infections (STIs), states make policy decisions that do not align with the evidence regarding the content and form of delivery—what is taught, where it is taught, and how it is taught. This has parallels to the pandemic, where across the United States, different states led by governors on varied sides of the political spectrum issued widely differing guidelines. Santelli and colleagues [49] write:

*“The lack of clear federal policy guidelines or resources for adolescent comprehensive sexuality education has resulted in a wide array of sex education policies at the state and school district level, and marked disparities by state and district in access to comprehensive sex education and sexual health outcomes”* (p. 276).

While we know that sex education contributes to physical, psychological, and sexual health [49], only 39 of 50 states mandate sex and/or HIV education, with 28 states mandating both [50]. Only 17 of these states require the program content to be medically accurate [50], which begs the question of why any state would permit false information to be conveyed in any educational program let alone sex education. This has obvious parallels to the way states have responded to the pandemic with various states mandating masks, and others ignoring scientific research supporting mask-wearing as a way to increase prevention. In the beginning, in March of 2020, journalists noted, “The last week laid bare a dizzying patchwork of local decision-making, as the largest quarantine in recent American history occurred in a juddering, piecemeal fashion” [51]. Some states closed bars, some restaurants, and some even prohibited walking together outside during the late fall surge of 2020. As with sex education, the choices governors made seemed to appeal to their constituency worried about work, the economy which would suffer from lockdowns, and tough images they attempted to maintain as leaders in areas where masculinist personas have greater sway (e.g., Governor Noem in South Dakota) [52]. When there is a national call for greater education and safety measures, and individual states are permitted policies that affect the entire nation, the protection of states’ rights is problematic. This is thus true for sex education, although rates of STIs and pregnancy do not present a national emergency. What would it mean, we ask, if adolescents received sex education that was nationally supported, education that the President and their cabinet endorsed as a public health initiative and linked to the advancement of health, well-being, and opportunity, especially for vulnerable populations? It would convey the message that we take care of each other, as a nation, to ensure that sex is voluntary, physically healthy, and a part of well-being.

### 4.3. Vulnerable Populations

All teens are treated as equally vulnerable; however, different groups, as in the pandemic, are differentially vulnerable. There is a disproportionate burden placed on girls, youths of color, teens with disabilities, and LGBT youths [53]. Youths who live in poverty have less access to sexual knowledge [54]. Black men report having received less sex education than other groups [55]. Latinx youth receive damaging messages [56]. Teens from the South and, in particular, from states that lack mandates regarding sex education or insist on abstinence education have higher teen pregnancies, births, and STIs [36].The African American teen birth rate in Texas was 44.1 out of 1000, compared to the national average of 29.4 and teen birth rates in Alabama, Arkansas, Louisiana, Oklahoma, and Texas are substantially higher among non-Hispanic Black individuals. The group most affected by HIV/AIDS and other STIs nationwide are Black Americans, and they are also the group least likely to have received information about this [37].

A neoliberal perspective might also suggest that the government and schools stay out of the business of educating about values. However, research shows that 75% of female teens report never having had any sex education by their parents. Over half of those who had education at home didn’t receive education about contraception or STIs [57].

## 5. Focus on Community: Four Requests

In this section, we give examples of how a community focus could be the foundation of sex education by exploring various ways in which sex education is taught and supported. Lessons from the pandemic call for a different kind of attitude and a different kind of sex education, namely one that looks for communal responsibility from issues like condom use to general sexual well-being. As we have been asked to wear masks to protect other people, we can ask teens with penises to wear condoms to protect someone else, states to mandate medically accurate information, individuals who hook up to try to understand what it might mean to the other person, and teens who use pornography to think about the lives of the people engaging in sex acts for their viewing. We address these four “asks” below.

### 5.1. Condom Distribution

Although some advocates of abstinence only education believe sex education should be a family affair, teens and their families may be hesitant to address the topic of condom use, as discussing sex and sexuality can be uncomfortable, and parents may believe that talking about condom use implies their approval, or that teens are or should be engaged in sexual activity [58]. In spite of the recurrent claim that sex education should happen within the family, there is widespread support for public sex education and one that encourages safe practices [59]. Still, within these efforts, statements such as “condom skills are taught to ensure that youth will be able to protect *themselves* (our emphasis) when they become sexually active” [60] highlights the primacy of the personal responsibility narrative common in such sex education. What becomes confusing is that, while teens are told to protect themselves (e.g., through condom usage), the conversation typically stops there, and condoms are unlikely to be provided in the context of such recommendations. While availability of condoms in spaces such as high schools may incite fear of encouraging more or earlier sexual activity, research shows that condom access programs at worst affect little change, and at best decrease rates of STIs and teen pregnancy, and increase safety in sexual situations [58]. This suggests positive outcomes of condom availability and is encouraging, but there is a continued absence of discussion around collective responsibility in the context of condom usage.

As with mask use, a collectivist approach could contribute to greater use of contraception which would lead to fewer teen pregnancies and STIs across the country. The community approach to condom use would suggest in the very least that we offer them in high schools. Currently, our schizophrenic messaging results in directives to use a condom, with no provision of them. We might also develop a discourse around condom use that discusses collective social goals of a society that protects and nurtures adolescents towards higher or further education and opportunities. Comfortability in discussing condom use and normalizing access to both boys and girls may also be important. Without contextualizing condom use within arguments about caring for other people, without also discussing power and gender dynamics, the reason to use condoms will always boil down to protecting oneself. This may be why Andrzejewski and colleagues [61] found that boys are more likely to take and use condoms when readily available in a school setting, whereas girls may feel uncomfortable doing so. While this may suggest boys are being thoughtful about whom they are having sex with, it is more likely that this also ties back to personal responsibility messaging, as boys are urged to use a condom in order to not get a girl pregnant. This is hardly a suggestion that encompasses a relational and non-heterosexist view of sex that we as a collective society might want to promote.

### 5.2. Medically Accurate Sex Education

Under a new administration that came into office on 20 January 2021, the U.S. has been learning in greater detail the way medically inaccurate information about the COVID-19 virus and treatment was supported and maintained by the previous President’s office and certain media outlets. Likewise, sex educators have long been concerned about medically inaccurate information, from falsehoods in early AOUM manuals that stated one could get HIV/AIDS from swimming pools [62,63], to the continued misrepresentations of the efficacy of condoms as an STI and pregnancy prevention strategy. Like the doctors speaking to the nation about COVID-19 [64], sex educators have also been concerned regarding restricting the distribution of information. Once President Biden took office, Dr. Anthony Fauci, who had been President Trump’s chief medical advisor, expressed to the New York Times, “The idea that you can get up here and talk about what you know—what the evidence, what the science is—and know that’s it, let the science speak... It is somewhat of a liberating feeling” [65]. Sex education hasn’t ever had a national acknowledgment and champion of the medically accurate science behind STIs and pregnancy, let alone more social issues such as rape and harassment. The closest we come to the assurance that information provided to adolescents is medically accurate is in the “mandates” that states have adopted [36]. Only 17 states require their program content to be medically accurate, and only nine states require the program to provide instruction that is appropriate for a student’s cultural background and that is not biased against any race, sex, or ethnicity [50]. What would it mean for a populace to share an understanding of pregnancy prevention and risk as one understands that washing one’s hands prevents colds and sneezing spreads them? Who has access to accurate information, and how?

### 5.3. Discussing Hookups

Sexual education, both formally and informally, is taught through gendered approaches [53,56,66,67]. Education around hookups is no exception. Elliott [43] in her discourse analysis of sex educators noted the way stereotypes of femininity and masculinity abounded when teachers discussed sexual relationships and restraining oneself (e.g., “men who can restrain themselves when the time comes are more manly!”). Women are charged with the responsibility for allowing or not allowing hookups to occur, through a lens of individual protection (e.g., “say *no* clearly,” “don’t drink too much,” etc.). While men may be “formally” told to “respect women,” they also receive messaging (i.e., from friends, family, media) that is antithetical to this and positions them to approach hookups as a game or competition in which “scoring” is the goal. This is problematic for a number of reasons, significantly for both the pressure and responsibility that women are given, and for its blatant disregard of the gender identity spectrum. But it’s also problematic in terms of its individual approach and focus on protection and consent.

In sex education, we teach teenagers about the dangers of hooking up and how drinking invalidates consent. Public discourse warns about hookups (i.e., people get hurt, obsessed, changed). A conservative approach would argue that sex is a powerful thing and that a teen isn’t equipped to handle all the feelings and responsibilities of it. A community approach might be instead that sex can be a powerful thing and so you want to make sure that, if you are pursuing pleasure or intimacy, you are not doing so at the expense of another. Advocating consent before hooking up reflects an individual rights contract model that assumes each person can take care of themself. But, similar to our argument around vulnerable populations, this idea implies that everyone has equal capacity and resources in order to take care of themselves. While there is nothing superficially wrong with talking about consequences and teaching consent, we are also missing an enormous piece of discourse around context: power dynamics, coercion across settings, the ethics of casual sex, and the vulnerabilities in other people that one should watch out and care for.

In parallel to the discussions above, we argue that we should be envisioning and setting goals for what collective and communitarian responsibility looks like when it comes to hookups and consent. There are real barriers to imagining collective responsibility, including our own biases and entrenched ideas about genders and personal responsibility, but also legal barriers—particularly in a punitive, carceral system, which is always looking for who to blame and punish. Communitarian responsibility implies that the “blame” lies somewhere in the system (or in the community) and that we are all complicit in matters of justice and injustice—and that is a hard idea to contend with.

### 5.4. Including Information about Pornography

The jury is in about the damaging effects of the reliance on and overuse of pornography as a means of sex education among youth [45]. And yet there is little that restricts the industry in the current context of free trade and understanding of freedoms, and, in many ways, themes of harm are acknowledged and reappropriated within porn media itself [68,69]. Furthermore, concerns related to pornography, such as videos of sexual abuse of minors that have run rampant on websites such as Pornhub, have been largely ignored by institutions that hold enough power to make a difference [70]. Protecting one’s child from these harms becomes a matter for individual parents, or the youths themselves [71]. As with the pandemic, if we think of pornography or the rape of women as something supported by institutions, and are focused on protecting individual freedoms rather than thinking of the good of the nation, protections will continue to be left to individuals and Big Porn/business will win out.

Alternatively, we could look to take a community approach, one founded in widespread consciousness-raising around human rights and objectification, sexism, violence against women, and the requirements of ethical sex. This view would recognize that porn may not be “dangerous” to the individual, but it is a public health risk that is damaging to society, and exploits those that are being traumatized for profit [70]. Therefore, we want to shift our approach from the individual (e.g., “Don’t watch porn, you’ll get addicted” or “Make better choices”) to the community and speak to ideals for society. This community approach would teach teens to think about porn from an ethical perspective, focused on community values, inclusive of those who watch, make, and sell porn. We would focus on the values of caring about others and not doing harm. Thus, if an individual is asked to practice self-restraint (as when an individual is asked to wear a mask), that individual does so not only for self but as a participant in a community of people who want to advance each other’s wellbeing.

## 6. Conclusions: Can Sex Education Learn from the Pandemic?

For those who have been working in areas where a community response and a caring attitude towards others would make a difference to the lives of individuals in need, that is, in the areas of public health, sex education, and public education, this moment of the pandemic, when citizens are asked to set aside individual needs and wishes, indeed freedoms, to think of the community and care about others’ health and well-being, is an important moment. The pandemic has made further explicit that a health crisis can only improve by combining a sense of caring for self with caring for others.

As shown in the examples above, sex education can and should take a more democratic, collective society approach. However, in the U.S., focusing on the well-being of others rather than just the self is understood as a kind of punishment, framed as a martyr’s giving up of individual rights for the good of the people. This has been made even more explicit during the COVID era. When there is a belief in absolute liberty for each individual, everything else feels like an imposition. This has led to little effort in considering the current opportunity to think differently about what it means to be a nation.

However, especially in sexual practice, thinking about the other person is central to health, well-being, and pleasure [3]. In sex education that is democratic, communal, and collective, all would learn medically accurate information about contraceptives, and their availability. Simple informational lessons would lead to discussions about inequities and context regarding gender, race, and ability, and how we as schools and communities aim to address these. To the extent risk is discussed in the classroom and to the extent there would be any fear-based messaging, the fear induced might be fear of harming others, or fear of reflecting poorly on their community. Pleasure messaging would be about pleasing the other and making someone else comfortable to have pleasure. Students would be allowed to investigate topics relating to sex in society, in order to become responsible citizens. They would learn what the social concerns are about pornography, rape, consent, sex work, objectification, hookups, and sexualization of children. If there is a lesson on “readiness” with regard to intercourse, the focus would be on the ramifications for all parties involved, and how one understands the effects of one’s own readiness on others. When a student is taught the mechanics of sex, they might hear about what could please the other and what can make another person feel uncomfortable. There would be discussions of the masculinist discourse about penis size. There would be the unpacking of what consent means legally and interpersonally, and how to spot lack of consent and step in, in situations where an assertive bystander may be needed. In Elliott’s words, “personal responsibility sex education should focus their lessons on social justice, unpacking how social inequalities are reproduced and how to interrupt them” [43] (p. 222), very much like Carmody’s [72] sexual ethics curriculum in New Zealand around dating and sexual violence and Lamb’s [73] Sexual Ethics for a Caring Society Curriculum in the U.S..

Public health should broaden its lens to create opportunities for sexual wellness and wellbeing in a population via community efforts. Sex education has lessons to learn from the pandemic response about how gender dynamics and a focus on the individual are not only bad for prevention but actually increase risk for everyone. Thus, we call on a public health response in the U.S. for government to mandate medically accurate sex education in every state, sex education that also includes social, relational, and civics lessons, and sex education that develops students’ knowledge and sense of justice around vulnerabilities of other people and groups they belong to. With new government leadership, this seems altogether possible. But neoliberalism pervades Western countries in ways that shape liberal governments, as well as the more conservative ones. If the pandemic has taught us anything, it is that we should all be in this together—for the good of everyone; and, yet, many have a way to go before they grasp this simple truth.
